# Supplementary material for: Geospatial variation and risk factors for malnutrition among postpartum women in rural Bangladesh
Source: PLOS Glob Public Health. 2026 Feb 6;6(2):e0005726. doi: 10.1371/journal.pgph.0005726 (PMC12880669; doi:10.1371/journal.pgph.0005726)
Supplement: S1 Text — (DOCX) [file pgph.0005726.s001.docx]

S1 Fig A. Summary of participant flow for analytic subsample

| **S1 Table A.** Baseline characteristics of women enrolled in Protein Plus trial that were included (n=3,801) and excluded (n=2,072) from this analysis. | | |
| --- | --- | --- |
|  | **Included (n=3,801)** | **Excluded (n=2,090)** |
| Individual Factors |  |  |
| Age | 24.74 ± 4.89 | 20.12 ± 5.36 |
| Number of previous live births | 1.67 ± 0.90 | 1.42 ± 0.90 |
| Maternal education |  |  |
| No Schooling | 500 (13.2) | 186 (9.3) |
| Class 1-9 | 2589 (68.1) | 1430 (71.5) |
| SSC Passed | 228 (6.0) | 117 (5.8) |
| 11 years + | 484 (12.7) | 262 (13.1) |
| Maternal occupation |  |  |
| No paid work | 2379 (62.6) | 1205 (60.3) |
| Own business | 1159 (30.5) | 394 (19.7) |
| Laborer | 96 (2.5) | 189 (9.5) |
| Private service | 118 (3.1) | 119 (6.0) |
| Farmer/sharecropper | 27 (0.7) | 64 (3.2) |
| Other | 22 (0.6) | 28 (1.4) |
| Household Factors |  |  |
| Household size | 4.40 ± 1.86 | 4.69 ± 2.15 |
| Living standard index | -0.22 ± 0.91 | -0.03 ± 0.92 |
| Values presented are mean ± SD or n (%). | | |

(n=2,072) from this analysis.

S1 Fig B. Semivariogram of BMI (continuous) at 6 months postpartum for women enrolled in Protein Plus


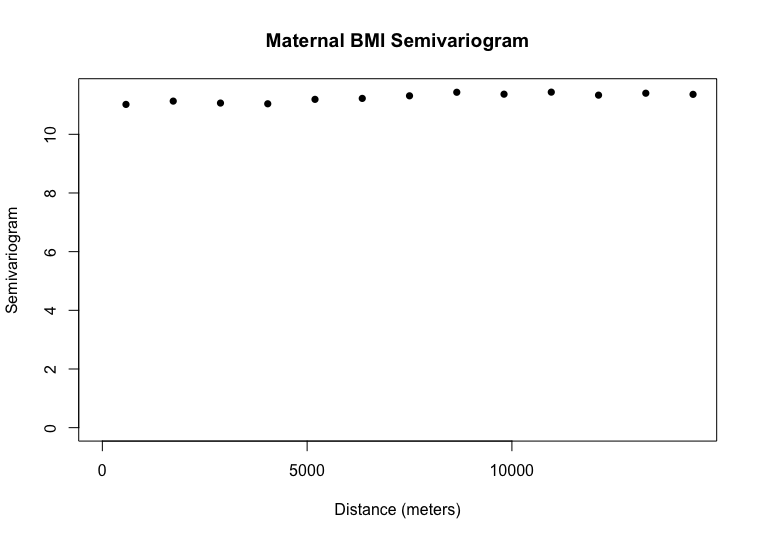


S1 Fig C. Difference in K functions for spatial intensity of underweight cases compared to normal weight controls among women enrolled in the Protein Plus trial at 6 months postpartum


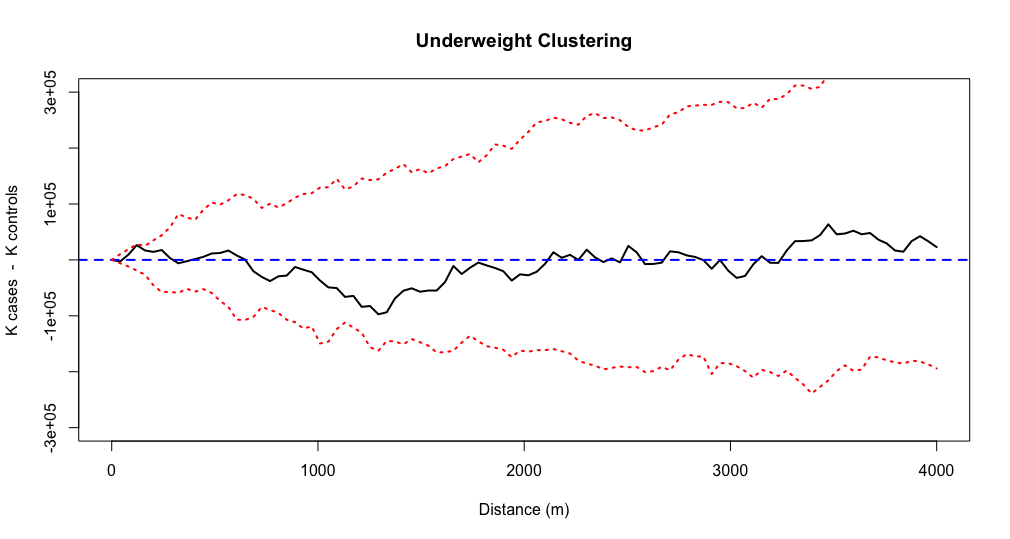


*Black line is difference in K functions for underweight cases vs controls. Red dotted line: Max & Min difference. Blue dotted line: expected under Ho (no difference).

S1 Fig D. Difference in K functions for spatial intensity of overweight cases compared to normal weight controls among women enrolled in the Protein Plus trial at 6 months postpartum


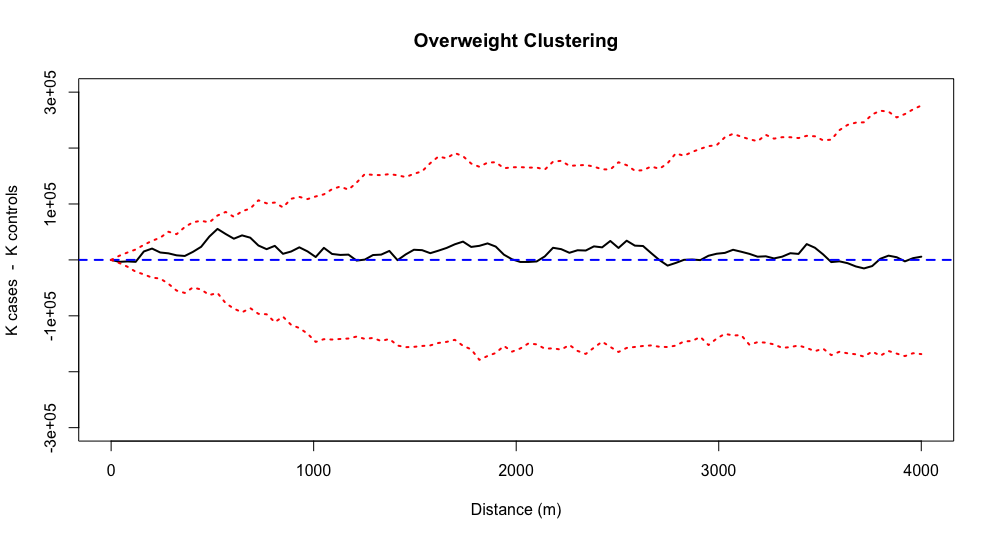


*Black line is difference in K functions for overweight cases vs controls. Red dotted line: Max & Min difference. Blue dotted line: expected under Ho (no difference).

S1 Figure E. Spatial intensity of underweight cases among women enrolled in the Protein Plus trial at 6 months postpartum


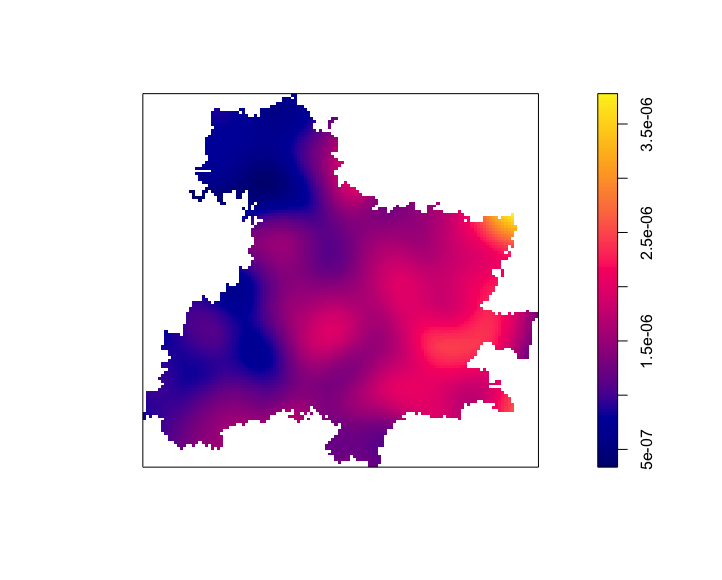


**S1 Fig F.** Spatial intensity of overweight cases among women enrolled in the Protein Plus trial at 6 months postpartum


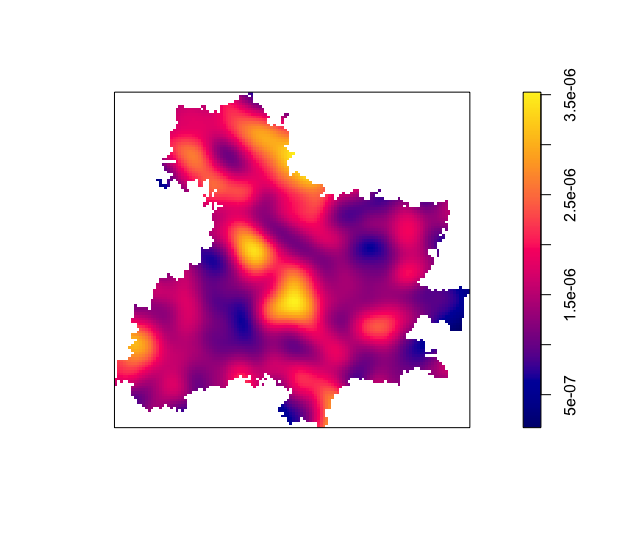


S1 Fig G. Prevalence of A) underweight (BMI <18.5 kg/m^2^) and B) overweight/obesity (BMI ≥ 25 kg/m^2^) at six months postpartum by mauza among women enrolled in the Protein Plus trial (n=3,801).


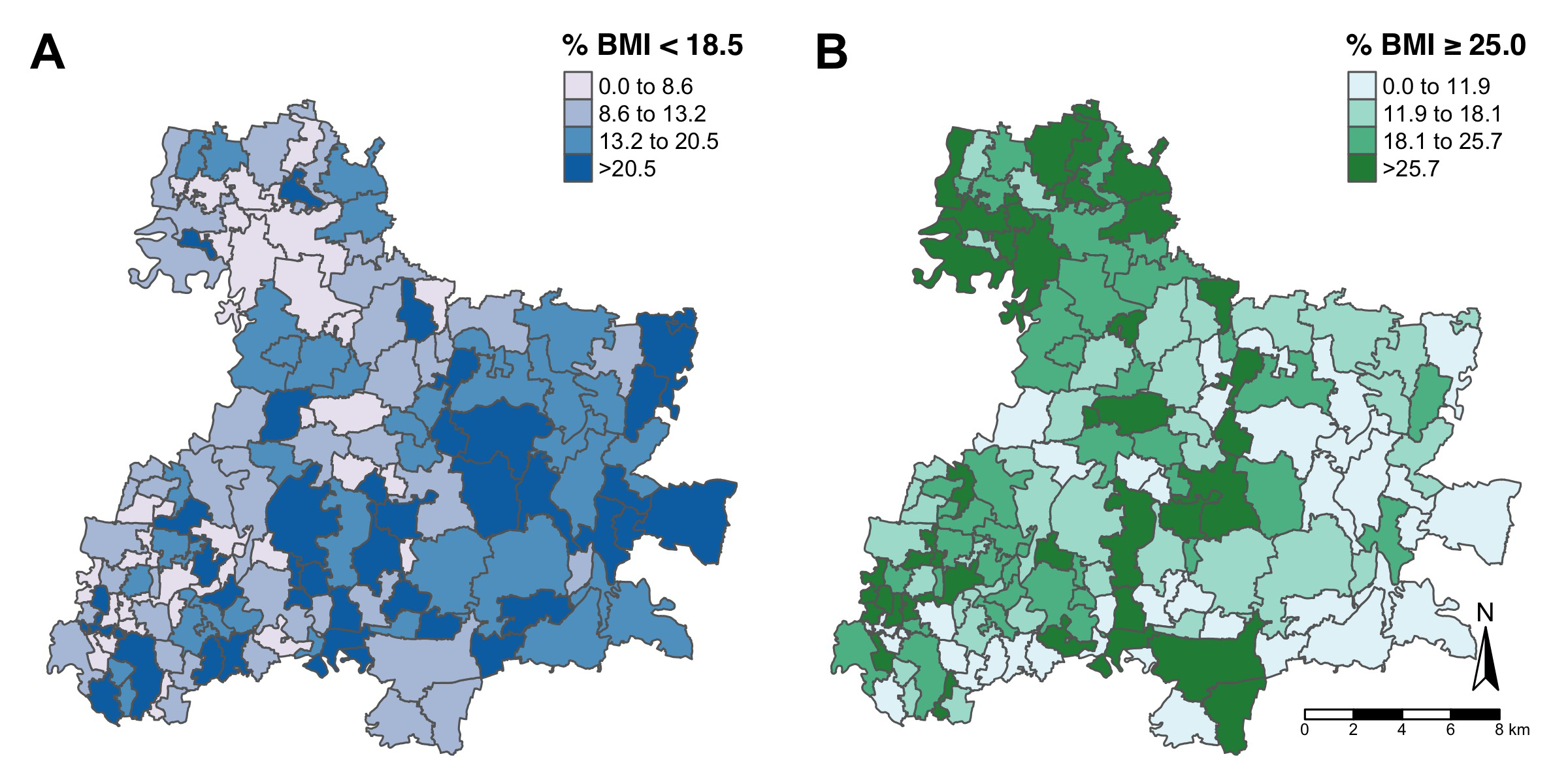


**cutoffs for each map are based on quartiles of prevalence A) underweight (BMI <18.5 kg/m^2^) and B) overweight/obesity (BMI ≥ 25 kg/m^2^)*

S1 Fig H. Moran’s I correlogram for prevalence of maternal underweight (BMI<18.5kg/m^2^)


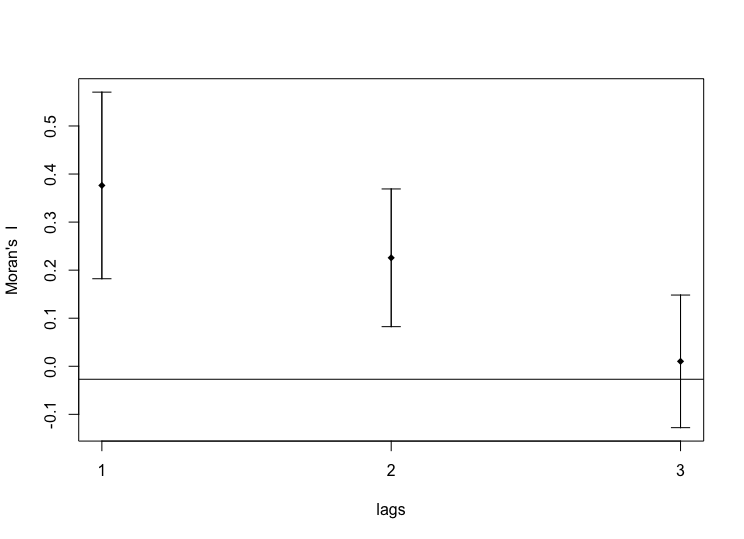


Confidence intervals for global Moran’s I were calculated using Monte Carlo methods, and boundaries are based on research site administrative units (Figure 1).

S1 Fig I. Moran's I correlogram for prevalence of maternal overweight (BMI≥25 kg/m^2^)


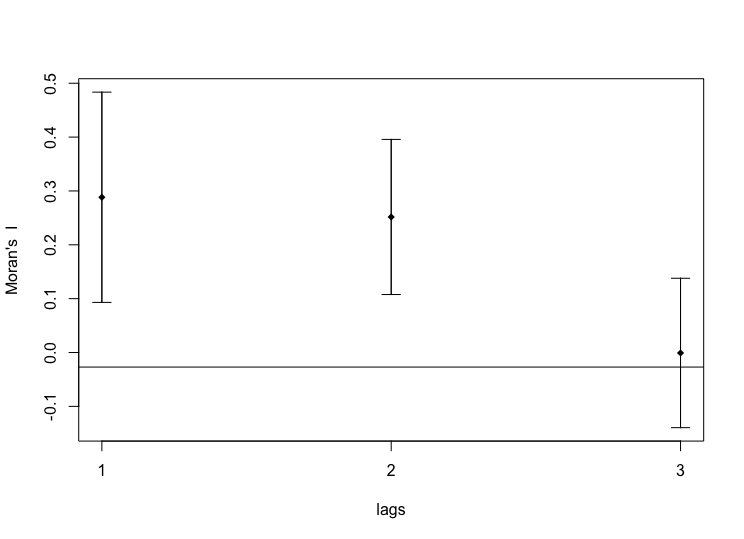


Confidence intervals for global Moran’s I were calculated using Monte Carlo methods, and boundaries are based on research site administrative units (Figure 1).

| **S1 Table B.** Relative risk ratios and 95% confidence intervals for the association between nutritional status and selected risk factors from bivariable multinomial logistic regression models among postpartum women in rural Bangladesh (n=3,801) | | | | |
| --- | --- | --- | --- | --- |
|  | **Underweight (BMI < 18.5 kg/m^2^) vs. Normal weight** | | **Overweight/obese (BMI ≥ 25.0 kg/m^2^) vs. Normal weight** | |
|  | **RRR (95% CI)** | **p-value** | **RRR (95% CI)** | **p-value** |
| Maternal Age (yrs) | 0.97 (0.95, 0.99) | <0.001 | 1.05 (1.03, 1.07) | <0.001 |
| 18-20 | Ref |  | Ref |  |
| 20-30 | 0.74 (0.60, 0.91) | <0.01 | 1.73 (1.36, 2.19) | <0.001 |
| 30-35 | 0.72 (0.51, 1.00) | 0.05 | 2.11 (1.53, 2.90) | <0.001 |
| 35+ | 0.59 (0.30, 1.14) | 0.12 | 2.57 (1.57, 4.22) | <0.001 |
| Number of Previous Live Births | 0.98 (0.87, 1.10) | 0.70 | 1.00 (0.90, 1.11) | 1.00 |
| Maternal Short Stature^a^ | 1.15 (0.92, 1.45) | 0.22 | 0.69 (0.54, 0.89) | <0.01 |
| Maternal Education |  |  |  |  |
| No Schooling | Ref |  | Ref |  |
| Class 1-9 | 1.00 (0.77, 1.30) | 1.00 | 1.15 (0.87, 1.52) | 0.32 |
| SSC Passed | 0.77 (0.48, 1.25) | 0.29 | 1.95 (1.30, 2.91) | <0.01 |
| 11 years+ | 0.90 (0.62, 1.30) | 0.57 | 2.25 (1.62, 3.13) | <0.001 |
| Food Variety Score^b^ | 0.96 (0.94, 0.99) | <0.01 | 1.09 (1.06, 1.11) | <0.001 |
| Less Healthy Food Consumption (>3 times/ week)^c^ | 0.76 (0.64, 0.92) | <0.01 | 1.35 (1.13, 1.60) | <0.001 |
| Market Density (1600m) |  |  |  |  |
| 0 to 2 markets | Ref |  | Ref |  |
| 3 to 4 markets | 0.86 (0.67, 1.10) | 0.23 | 0.80 (0.62, 1.02) | 0.08 |
| 5 to 6 markets | 0.79 (0.61, 1.02) | 0.07 | 0.90 (0.70, 1.16) | 0.41 |
| > 6 markets | 0.71 (0.54, 0.94) | 0.02 | 0.95 (0.74, 1.23) | 0.71 |
| Variety Store Density (400m) |  |  |  |  |
| 0 to 1 variety stores | Ref |  | Ref |  |
| 2 to 3 variety stores | 0.94 (0.71, 1.26) | 0.70 | 0.88 (0.67, 1.15) | 0.35 |
| 4 to 5 variety stores | 0.90 (0.68, 1.20) | 0.49 | 0.80 (0.61, 1.05) | 0.11 |
| 6 ≥ variety stores | 0.92 (0.70, 1.22) | 0.57 | 0.92 (0.72, 1.20) | 0.55 |
| LSI Quintile |  |  |  |  |
| Q1 (lowest) | Ref |  | Ref |  |
| Q2 | 0.72 (0.55, 0.93) | 0.01 | 1.11 (0.80, 1.54) | 0.54 |
| Q3 | 0.56 (0.42, 0.74) | <0.001 | 1.66 (1.22, 2.25) | <0.01 |
| Q4 | 0.64 (0.48, 0.83) | <0.01 | 1.85 (1.37, 2.50) | <0.001 |
| Q5 (Highest) | 0.53 (0.40, 0.71) | <0.001 | 3.02 (2.26, 4.03) | <0.001 |
| Food Security Status |  |  |  |  |
| Food Secure | Ref |  | Ref |  |
| Mild to Moderate Food Insecurity | 0.94 (0.77, 1.14) | 0.52 | 0.87 (0.72, 1.05) | 0.16 |
| Severe Food Insecurity | 1.07 (0.65, 1.77) | 0.78 | 0.45 (0.23, 0.88) | 0.02 |
| Season |  |  |  |  |
| Autumn | Ref |  | Ref |  |
| Early Monsoon | 1.30 (0.38, 4.51) | 0.67 | 1.60 (0.46, 5.54) | 0.46 |
| Late Monsoon | 1.15 (0.33, 3.98) | 0.83 | 1.53 (0.44, 5.30) | 0.50 |
| Spring | 1.39 (0.40, 4.80) | 0.61 | 1.31 (0.38, 4.55) | 0.67 |
| Summer | 1.45 (0.40, 4.80) | 0.61 | 1.59 (0.46, 5.51) | 0.46 |
| Winter | 1.02 (0.29, 3.59) | 0.97 | 1.10 (0.31, 3.87) | 0.88 |
| Ramadan | 1.40 (1.07, 1.83) | 0.01 | 1.28 (0.99, 1.66) | 0.06 |
| RRR: Relative risk ratio, SSC: Secondary school certificate, FVS: food variety score, LSI: Living standard index. Normal weight is defined as BMI≥18.5 kg/m^2^ & BMI<25 kg/m^2^ ^a^ Short stature defined as maternal height< 145cm.  ^b^ Food variety scores are defined as the average number of non-starchy staple foods items or groups consumed in the last week excluding sweet & salty snacks and sugary sweetened beverages. Scores range from 1-25. ^c^ Less healthy food consumption is defined as consumption of less healthy food options at least 3 times on average in the 7-day recall period. Less healthy foods options were defined as soda, sweet yogurt, sugar cane, cake/biscuits, mishti, chocolate/candy, ice cream, salty snacks, and food fried in oil | | | | |

Sup

| **S1 Table C.** Relative risk ratios and 95% confidence intervals for the association between nutritional status and selected risk factors from multivariable multinomial logistic regression models among postpartum women in rural Bangladesh (n=3,801) | | | | |  |
| --- | --- | --- | --- | --- | --- |
|  | **Underweight  (BMI < 18.5 kg/m^2^) vs. Normal weight** | | **Overweight/obese  (BMI ≥ 25.0 kg/m^2^) vs. Normal weight** | |  |
|  |  |  |  |  |  |
|  | **RRR (95% CI)** | **p-value** | **RRR (95% CI)** | **p-value** |  |
| Maternal Age | 0.96 (0.94, 0.98) | <0.001 | 1.06 (1.04, 1.08) | <0.001 |  |
| Maternal Short Stature^a^ | 1.09 (0.86, 1.37) | 0.47 | 0.82 (0.64, 1.06) | 0.13 |  |
| Maternal Education |  |  |  |  |  |
| No Schooling | Ref |  | Ref |  |  |
| Class 1-9 | 1.06 (0.80, 1.41) | 0.68 | 1.10 (0.81, 1.49) | 0.56 |  |
| SSC Passed | 0.98 (0.58, 1.65) | 0.93 | 1.37 (0.88, 2.15) | 0.17 |  |
| 11 years+ | 1.19 (0.77, 1.82) | 0.44 | 1.53 (1.03, 2.27) | 0.04 |  |
| Food Variety Score^b^ (quartiles) |  |  |  |  |  |
| Q1 (Lowest) | Ref |  | Ref |  |  |
| Q2 | 0.97 (0.76, 1.25) | 0.84 | 0.87 (0.66, 1.15) | 0.34 |  |
| Q3 | 1.00 (0.77, 1.29) | 0.97 | 1.34 (1.02, 1.75) | 0.03 |  |
| Q4 (Highest) | 0.98 (0.73, 1.30) | 0.87 | 1.38 (1.04, 1.83) | 0.02 |  |
| Less Healthy Food Consumption  (>3 times/ week)^c^ | 0.82 (0.67, 1.00) | 0.05 | 0.97 (0.80, 1.18) | 0.78 |  |
|  |  |  |  |  |  |
| Market Density (1600m) |  |  |  |  |  |
| 0 to 2 markets | Ref |  | Ref |  |  |
| 3 to 4 markets | 0.86 (0.66, 1.11) | 0.23 | 0.81 (0.62, 1.05) | 0.11 |  |
| 5 to 6 markets | 0.80 (0.61, 1.04) | 0.09 | 0.90 (0.69, 1.16) | 0.40 |  |
| > 6 markets | 0.72 (0.55, 0.95) | 0.02 | 0.91 (0.70, 1.18) | 0.48 |  |
| LSI Quintile |  |  |  |  |  |
| Q1 (Lowest) | Ref |  | Ref |  |  |
| Q2 | 0.70 (0.54, 0.91) | <0.01 | 1.08 (0.78, 1.51) | 0.64 |  |
| Q3 | 0.52 (0.39, 0.70) | <0.001 | 1.56 (1.14, 2.14) | <0.01 |  |
| Q4 | 0.60 (0.45, 0.80) | <0.001 | 1.66 (1.20, 2.29) | <0.01 |  |
| Q5 (Highest) | 0.49 (0.34, 0.69) | <0.001 | 2.37 (1.69, 3.32) | <0.001 |  |
| Food Security Status |  |  |  |  |  |
| Food Secure | Ref |  | Ref |  |  |
| Mild to Moderate Food Insecurity | 0.85 (0.69, 1.04) | 0.12 | 1.04 (0.86, 1.27) | 0.67 |  |
| Severe Food Insecurity | 0.85 (0.50, 1.43) | 0.53 | 0.71 (0.36, 1.41) | 0.33 |  |
| Ramadan | 1.46 (1.11, 1.91) | <0.01 | 1.21 (0.92, 1.58) | 0.17 |  |
| RRR: Relative risk ratio, SSC: Secondary school certificate, FVS: food variety score, LSI: Living standard index. Normal weight is defined as BMI≥18.5 kg/m^2^ & BMI<25 kg/m^2^ Models included the following covariates: maternal age (continuous), maternal short stature (categorical, reference= ≥145cm), maternal education (categorical, reference= “No schooling”), food variety scores (categorical, reference= lowest quartile), less healthy food consumption (categorical, reference= >3 times/week), market density (categorical, reference = 0 to 2 markets), living standard index (categorical, reference= lowest quartile), food insecurity (categorical, reference = food secure) and 6 month maternal survey conducted during Ramadan (binary, reference=no).  ^a^ Short stature defined as maternal height< 145cm.  ^b^ Food variety scores are defined as the average number of non-starchy staple foods items or groups consumed in the last week excluding sweet & salty snacks and sugary sweetened beverages. Scores range from 1-25. ^c^ Less healthy food consumption is defined as consumption of less healthy food options at least 3 times on average in the 7-day recall period. Less healthy foods options were defined as soda, sweet yogurt, sugar cane, cake/biscuits, mishti, chocolate/candy, ice cream, salty snacks, & food fried in oil | | | | |  |

| **S1 Table D.** Relative risk ratios and 95% confidence intervals for the association between nutritional status (using Asian-specific BMI cutoffs*) and selected risk factors from bivariate multinomial logistic regression models among postpartum women in rural Bangladesh (n=3,801)**.** | | | | |  |
| --- | --- | --- | --- | --- | --- |
|  | **Underweight  (BMI < 18.5 kg/m^2^) vs. Normal weight** | | **Overweight/obese  (BMI ≥ 23.0 kg/m^2^) vs. Normal weight** | |  |
|  |  |  |  |  |  |
|  | **RRR (95% CI)** | **p-value** | **RRR (95% CI)** | **p-value** |  |
| Maternal Age (yrs) | 0.97 (0.96, 0.99) | 0.01 | 1.05 (1.03, 1.06) | <0.001 |  |
| 18-20 | Ref |  | Ref |  |  |
| 20-30 | 0.85 (0.69, 1.04) | 0.12 | 1.95 (1.62, 2.35) | <0.001 |  |
| 30-35 | 0.78 (0.55, 1.10) | 0.15 | 1.94 (1.48, 2.53) | <0.001 |  |
| 35+ | 0.69 (0.35, 1.35) | 0.28 | 2.56 (1.65, 3.93) | <0.001 |  |
| Number of Previous Live Births | 0.96 (0.85, 1.08) | 0.52 | 0.96 (0.88, 1.05) | 0.36 |  |
| Maternal Short Stature^a^ | 1.05 (0.84, 1.33) | 0.66 | 0.64 (0.52, 0.78) | <0.001 |  |
| Maternal Education |  |  |  |  |  |
| No Schooling | Ref |  | Ref |  |  |
| Class 1-9 | 1.00 (0.77, 1.32) | 0.98 | 1.08 (0.87, 1.35) | 0.47 |  |
| SSC Passed | 0.82 (0.50, 1.34) | 0.42 | 1.66 (1.18, 2.34) | <0.01 |  |
| 11 years+ | 1.06 (0.73, 1.56) | 0.75 | 2.26 (1.71, 2.98) | <0.001 |  |
| Food Variety Score^b^ | 0.98 (0.95, 1.00) | 0.07 | 1.08 (1.06, 1.10) | <0.001 |  |
| Less Healthy Food Consumption (>3 times/ week)^c^ | 0.80 (0.66, 0.97) | 0.02 | 1.32 (1.14, 1.52) | <0.001 |  |
| Market Density (1600m) |  |  |  |  |  |
| 0 to 2 markets | Ref |  | Ref |  |  |
| 3 to 4 markets | 0.86 (0.66, 1.11) | 0.25 | 0.89 (0.72, 1.10) | 0.27 |  |
| 5 to 6 markets | 0.77 (0.59, 1.01) | 0.06 | 0.89 (0.72, 1.10) | 0.29 |  |
| > 6 markets | 0.74 (0.56, 0.98) | 0.04 | 1.07 (0.86, 1.32) | 0.56 |  |
| Grocery Shop Density (400m) |  |  |  |  |  |
| 0 to 1 grocery shops | Ref |  | Ref |  |  |
| 2 to 3 grocery shops | 0.97 (0.72, 1.31) | 0.84 | 1.00 (0.80, 1.26) | 0.99 |  |
| 4 to 5 grocery shops | 0.91 (0.68, 1.22) | 0.54 | 0.91 (0.73, 1.15) | 0.43 |  |
| 6 ≥ grocery shops | 0.93 (0.70, 1.24) | 0.64 | 0.99 (0.80, 1.23) | 0.94 |  |
| LSI Quintile |  |  |  |  |  |
| Q1 (Lowest) | Ref |  | Ref |  |  |
| Q2 | 0.74 (0.57, 0.97) | 0.03 | 1.17 (0.92, 1.50) | 0.20 |  |
| Q3 | 0.62 (0.46, 0.82) | <0.001 | 1.71 (1.35, 2.17) | <0.001 |  |
| Q4 | 0.70 (0.53, 0.93) | 0.01 | 1.83 (1.44, 2.32) | <0.001 |  |
| Q5 (Highest) | 0.64 (0.47, 0.86) | <0.01 | 2.86 (2.26, 3.61) | <0.001 |  |
| Food Security Status |  |  |  |  |  |
| Food Secure | Ref |  | Ref |  |  |
| Mild to Moderate Food Insecurity | 0.93 (0.76, 1.13) | 0.46 | 0.90 (0.77, 1.05) | 0.19 |  |
| Severe Food Insecurity | 1.08 (0.64, 1.81) | 0.77 | 0.72 (0.46, 1.12) | 0.15 |  |
| Season |  |  |  |  |  |
| Autumn | Ref |  | Ref |  |  |
| Early Monsoon | 1.15 (0.32, 4.22) | 0.84 | 0.91 (0.37, 2.21) | 0.83 |  |
| Late Monsoon | 0.96 (0.26, 3.51) | 0.95 | 0.79 (0.32, 1.93) | 0.61 |  |
| Spring | 1.10 (0.30, 4.00) | 0.89 | 0.62 (0.25, 1.52) | 0.30 |  |
| Summer | 1.21 (0.33, 4.40) | 0.77 | 0.79 (0.32, 1.94) | 0.61 |  |
| Winter | 0.86 (0.23, 3.16) | 0.81 | 0.67 (0.27, 1.65) | 0.38 |  |
| Ramadan | 1.41 (1.07, 1.86) | 0.01 | 1.17 (0.93, 1.46) | 0.18 |  |
| RRR: Relative risk ratio, SSC: Secondary school certificate, FVS: food variety score, LSI: Living standard index. *Normal weight is defined as BMI≥18.5 kg/m^2^ & BMI<23 kg/m^2^ ^a^ Short stature defined as maternal height< 145cm.  ^b^ Food variety scores are defined as the average number of non-starchy staple foods items or groups consumed in the last week excluding sweet & salty snacks and sugary sweetened beverages. Scores range from 1-25. ^c^ Less healthy food consumption is defined as consumption of less healthy food options at least 3 times on average in the 7-day recall period. Less healthy foods options were defined as soda, sweet yogurt, sugar cane, cake/biscuits, mishti, chocolate/candy, ice cream, salty snacks & food fried in oil | | | | |  |

| **S1 Table E.** Relative risk ratios and 95% confidence intervals for the association between nutritional status (using Asian-specific BMI cutoffs*) and selected risk factors from multivariate multinomial logistic regression models among postpartum women in rural Bangladesh (n=3,801) | | | | |  |
| --- | --- | --- | --- | --- | --- |
|  | **Underweight  (BMI < 18kg/m^2^) vs. Normal weight** | | **Overweight/obese  (BMI ≥ 23.0 kg/m^2^) vs. Normal weight** | |  |
|  |  |  |  |  |  |
|  | **RRR (95% CI)** | **p-value** | **RRR (95% CI)** | **p-value** |  |
| Maternal Age | 0.97 (0.95, 0.99) | <0.01 | 1.06 (1.04, 1.08) | <0.001 |  |
| Maternal Short Stature^a^ | 1.02 (0.80, 1.29) | 0.90 | 0.72 (0.59, 0.89) | <0.01 |  |
| Maternal Education |  |  |  |  |  |
| No Schooling | Ref |  | Ref |  |  |
| Class 1-9 | 1.06 (0.79, 1.42) | 0.68 | 1.05 (0.82, 1.33) | 0.71 |  |
| SSC Passed | 0.99 (0.58, 1.69) | 0.98 | 1.23 (0.84, 1.81) | 0.28 |  |
| 11 years+ | 1.33 (0.86, 2.06) | 0.20 | 1.63 (1.17, 2.27) | <0.01 |  |
| Food Variety Score^b^ (quartiles) |  |  |  |  |  |
| Q1 (Lowest) | Ref |  | Ref |  |  |
| Q2 | 0.97 (0.75, 1.25) | 0.81 | 0.92 (0.74, 1.15) | 0.47 |  |
| Q3 | 0.98 (0.75, 1.28) | 0.89 | 1.12 (0.90, 1.39) | 0.31 |  |
| Q4 (Highest) | 1.01 (0.75, 1.36) | 0.95 | 1.28 (1.02, 1.61) | 0.04 |  |
| Less Healthy Food Consumption  (>3 times/ week)^c^ | 0.82 (0.67, 1.01) | 0.06 | 1.01 (0.86, 1.18) | 0.93 |  |
|  |  |  |  |  |  |
| Market Density (1600m) |  |  |  |  |  |
| 0 to 2 markets | Ref |  | Ref |  |  |
| 3 to 4 markets | 0.86 (0.66, 1.12) | 0.25 | 0.90 (0.73, 1.12) | 0.34 |  |
| 5 to 6 markets | 0.78 (0.59, 1.02) | 0.07 | 0.89 (0.71, 1.10) | 0.27 |  |
| > 6 markets | 0.74 (0.56, 0.99) | 0.04 | 1.04 (0.83, 1.29) | 0.75 |  |
| LSI Quintile |  |  |  |  |  |
| Q1 (Lowest) | Ref |  | Ref |  |  |
| Q2 | 0.72 (0.55, 0.95) | 0.02 | 1.18 (0.91, 1.52) | 0.21 |  |
| Q3 | 0.58 (0.43, 0.78) | <0.001 | 1.69 (1.32, 2.17) | <0.001 |  |
| Q4 | 0.66 (0.49, 0.89) | <0.01 | 1.72 (1.33, 2.23) | <0.001 |  |
| Q5 (Highest) | 0.56 (0.39, 0.71) | <0.01 | 2.32 (1.75, 3.06) | <0.001 |  |
| Food Security Status |  |  |  |  |  |
| Food Secure | Ref |  | Ref |  |  |
| Mild to Moderate Food Insecurity | 0.87 (0.70, 1.07) | 0.18 | 1.08 (0.92, 1.27) | 0.35 |  |
| Severe Food Insecurity | 0.91 (0.53, 1.55) | 0.73 | 1.11 (0.69, 1.77) | 0.67 |  |
| Ramadan | 1.45 (1.10, 1.92) | <0.01 | 1.10 (0.87, 1.39) | 0.42 |  |
| RRR: Relative risk ratio, SSC: Secondary school certificate, FVS: food variety score, LSI: Living standard index. Normal weight is defined as BMI≥18.5 kg/m^2^ & BMI<23 kg/m^2^ Models included the following covariates: maternal age (continuous), maternal short stature (categorical, reference= ≥145cm), maternal education (categorical, reference= “No schooling”), food variety scores (categorical, reference= lowest quartile), less healthy food consumption (categorical, reference = >3 times/week), market density (categorical, reference = 0 to 2 markets), living standard index (categorical, reference= lowest quartile), food insecurity (categorical, reference = food secure) and 6 month maternal survey conducted during Ramadan (binary, reference=no).  ^a^ Short stature defined as maternal height< 145cm.  ^b^ Food variety scores are defined as the average number of non-starchy staple foods items or groups consumed in the last week excluding sweet & salty snacks and sugary sweetened beverages. Scores range from 1-25. ^c^ Less healthy food consumption is defined as consumption of less healthy food options at least 3 times on average in the 7-day recall period. Less healthy foods options were defined as soda, sweet yogurt, sugar cane, cake/biscuits, mishti, chocolate/candy, ice cream, salty snacks & food fried in oil | | | | |  |

**S1 Fig J**. Semivariogram of residuals from linear regression model assessing relationship between BMI (continuous) and selected risk factors


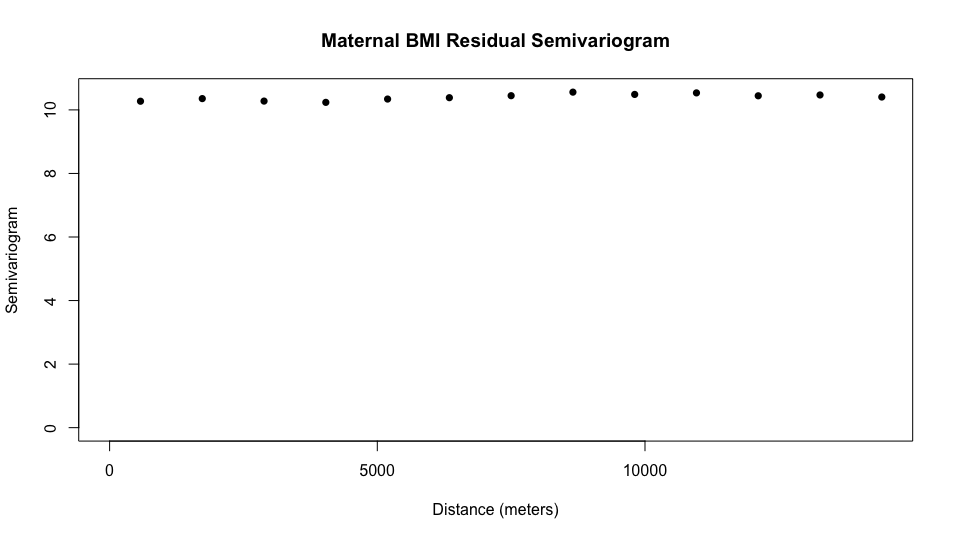


*selected risk factors include: maternal age, maternal education, food variety score, above average less healthy food consumption, market availability, household SES, and household food security status
