# Supplementary material for: Geospatial variation and risk factors for malnutrition among postpartum women in rural Bangladesh
Source: PLOS Glob Public Health. 2026 Feb 6;6(2):e0005726. doi: 10.1371/journal.pgph.0005726 (PMC12880669; doi:10.1371/journal.pgph.0005726)
Supplement: Attachment — Submitted filename: Responses to Reviewer.docx [file pgph.0005726.s003.docx]

# Reviewer #1:

Dear Review 1,

Thank you for taking the time to review our manuscript. We appreciate your input and hope that the changes we made to our manuscript help clarify our methods and overall conclusions. Some comments below (points 1-4) appear to reference the abstract. Unfortunately, with a 300 word limit in the abstract we are not able to address all these points in the abstract. In these instances, we have reviewed the main text to ensure that these points are addressed or further clarified. Line numbers refers to the clean version of the manuscript.

**Abstract**
1. Enhance the Spatial Analysis Component
Clearly define how geospatial variation was measured and analyzed.
Provide details on the spatial clustering methodology (e.g., Moran’s I, spatial regression models).
Explain why no spatial dependence was found at the household level but observed at the community level.

In the abstract we have now added the spatial clustering methodology to the sentence on geospatial variation on **lines 8-9.**

“We assessed geospatial variation in nutritional status at the individual level using point pattern analysis and at the spatial area level using global Moran’s I.”

Further details on spatial clustering methodology can be found on **lines 139-156** in the methods section of the full text.

In the discussion section on **lines 316-322** we have clarified text to include our explanation of why there was no spatial dependence more prominent.

“Within the JiVitA study site, we found geospatial variation in nutritional status at the area level, although at the household level, we found no evidence of geospatial variation. The lack of statistically significant spatial dependence at the household level is most likely due to high population density and variability in nutritional status among neighboring women within the same area unit. At the area level, we see a higher prevalence of undernutrition in the eastern quadrant of the research site and a higher prevalence of overweight and obesity in the northern and western quadrants. Few mauzas have a high prevalence of both under and overnutrition. “

2. Clarify the Methodological Approach
Justify the use of multinomial logistic regression and discuss its limitations.
Address potential biases, including sample selection and confounding variables.
Specify whether the Protein Plus trial design influenced the study's generalizability.

Thank you for this comment. In the main text, we have reorganised the methods section and added text to better justify the use of multinomial logistic regression. On **lines 160-162**, we have added the sentence

“We selected a multinomial model to allow our outcome of interest to have three categories: underweight (BMI <18.5 kg/m^2^ ), normal weight (BMI: 18.5-24.9 kg/m^2^) and overweight/obese (BMI ≥ 25.0 kg/m^2^).”

In the discussion section of the main text we have address study’s generalizability on **lines 300-315**

“Although our results are from an area of Bangladesh with many nutrition-related interventions and studies (28,43,44), the prevalences of undernutrition and overweight/obesity in this study population are similar to other studies from rural Bangladesh, indicating that results from our risk factor analysis may be generalizable to similar populations in rural areas in Bangladesh. The average BMI in our study population was 21.8 kg/m2, which is consistent with modeled estimates of BMI in rural Bangladesh from 2017 (3).”

We have also added a sentence on limitations of our regression model and confounding variables on **lines 387-390** in the discussion.

“Finally, confounders included in our multinomial model were limited to the data available; therefore, our models may be missing key risk factors and susceptible to residual confounding. As this is a cross-sectional analysis, we are only able to assess association rather than causation.”

3. Improve Contextualization and Interpretation
Compare findings with other studies on malnutrition in similar rural South Asian settings.
Discuss broader implications, particularly how these findings can inform public health policies.
Explain the mechanisms by which education and socioeconomic status influence nutritional status.

Unfortunately, given the 300-word limit of an abstract, we are not able to get into this detail in the abstract. We do agree that these are important and address each of these points in detail in the discussion section

4. Strengthen the Conclusion
Move beyond simply stating the double burden of malnutrition—propose potential interventions.
Discuss how policymakers or healthcare providers can address these disparities.
Acknowledge study limitations (e.g., self-reported dietary intake, potential measurement errors).

Unfortunately, given the 300-word limit of an abstract, we are not able to get into this detail in the abstract. We do agree that these are important and address each of these points in detail in the discussion section. We have noted in the discussion rather than proposing specific interventions, we encourage geospatial monitoring and evaluation of interventions.

1. Improve Clarity and Structure
The section contains dense information that could be better structured for readability. Breaking it into subsections (e.g., study site, participant selection, ethical considerations) would enhance clarity.

Thank you for this comment and we have broken up the methods section to include the following subsections: Study Area, Participant Selection, Ethical Approval, Data Collection, and Statistical analysis.

Define JiVitA Research Site at the beginning instead of mentioning it mid-sentence.

We have revised the introduction to the JiVitA Research Site based on your comments on **lines 73-76.**

“Data for this analysis were collected at the JiVitA Research Site located in the Gaibandha District in Northwestern Bangladesh. This research site was established in 2000 by researchers at Johns Hopkins University (28). The research site encompasses a rural geographic area of approximately 435km^2^, with a population of approximately 650,000 individuals.”

2. Justify Study Site Selection
The authors should explain why Gaibandha district was chosen for the study.
Provide context on the malnutrition burden in this area compared to other regions in Bangladesh.

We have added text (see point above) regarding the research site to justify why the Gaibandha district was chosen for the study. In the discussion section on **lines 300-311** we compared malnutrition burden in this area compared to other regions in Bangladesh.

“Although our results are from an area of Bangladesh with many nutrition-related interventions and studies (28,43,44), the prevalences of undernutrition and overweight/obesity in this study population are similar to other studies from rural Bangladesh, indicating that results from our risk factor analysis may be generalizable to similar populations in rural areas in Bangladesh. The average BMI in our study population was 21.8 kg/m2, which is consistent with modeled estimates of BMI in rural Bangladesh from 2017 (3). On the other hand, in comparison with the 2017–2018 Demographic and Health Survey (DHS), we found a higher prevalence of underweight and a lower prevalence of overweight and obesity among ever-married women in rural areas. Estimates from the DHS indicate that 13% of ever-married women were classified as underweight based on BMI, while 28% of ever-married women were classified as overweight or obese based on BMI in rural areas (45). Another study that explored risk factors of nutritional status using anthropometric data from 2011 in the Sylhet Division of Bangladesh estimated a significantly higher percentage of underweight women (37.0%) and a lower prevalence of overweight and obesity (7.2%) compared to our study (25). This is consistent with findings from DHS, which indicate that the Sylhet Division has the highest prevalence of undernutrition for ever-married women (45). Differences may also be attributable to temporal trends in BMI between studies. Studies that have looked at the change in BMI in Bangladesh have shown that the average BMI is rising most rapidly in rural areas of the country (3,46,47).”

3. Address Selection Bias
The eligibility criteria (women must be married and live with husbands) exclude single mothers or women in other family structures, which could introduce bias.
Discuss how this exclusion might affect the generalizability of findings to all postpartum women in rural Bangladesh.

Thank you for this comment and it was one we discussed among co-authors during the planning of the statistical analysis. The cultural and religious contexts in this rural region make pregnancy before/without marriage extremely prohibited. Therefore, the unmarried pregnancy and pregnancy with not living husband are extremely rare and/or not disclosed. Given the cultural norms in rural Bangladesh, we do not believe this would significantly impact the representativeness of our study. It is likely that unmarried women who are pregnant are of lower SES and therefore potentially at higher risk of being classified as underweight 6 months postpartum.

4. Ethical Considerations
While approvals are mentioned, it's unclear whether informed consent was obtained from participants for this specific analysis, not just for the primary trials (Protein Plus and mCARE-II).
Did women have the option to withdraw from the study after enrollment in pregnancy surveillance?

Yes, they would have had the option to withdraw from pregnancy surveillance under the mCARE-II protocol. In the consent forms, the analysis that will be conducted are not specified. We asked for permission to collect certain information such as anthropometry, diet, and health.

5. Clarify Data Collection Timeline
The study mentions data collection at three months postpartum, but it’s unclear if all women were enrolled at the same time or if enrollment was rolling.
Specify if seasonality in food availability or health conditions could have influenced nutritional outcomes.

Thank you for this comment. Women were enrolled on a rolling basis. We have updated a sentence in the participation selection part of the methods to read on **lines 80-82.**

“The Protein Plus trial collected data on a rolling basis from women and infants starting at three months postpartum from 01 September 2018 to 31 March 2020.”

Seasonality could impact diet and nutrition (although we mention later in the text that diet was measured across seasons to account for seasonal variation). We included seasonality as a risk factor in our multinomial analysis (**lines 188-190**). Seasonality was not a significant risk factor in either bivariate of multivariate analysis (**Supplemental table 2 and 3).**

1. Improve Consistency and Completeness in Anthropometric Measurements
Height was measured three times, while weight was measured only once—why the inconsistency? Weight should also be measured multiple times to reduce measurement error.

Measuring height is more susceptible to error due to posture and measurement technique. Three measurements were taken to ensure accuracy and is standard in anthropometry surveys. While weight may fluctuate, there is significantly less measurement error in the short term which results in weight typically being measured only once by the data collector. All anthropometry data collectors were highly trained and supervised by the quality control team throughout the data collection process. We have added more information in the methods section on **lines 100-105.**

“Trained fieldworkers collected data on maternal anthropometric measures at three and six months postpartum at the household. Prior to data collection, field workers received a refresher training on standard anthropometry protocols and were required to achieve a target of 1.0% and 1.5% for inter-observer and intra-observer technical errors of measurement, respectively. Additionally, field workers received regular supervision from the quality control team throughout data collection.”

Clarify why height was measured at three months but weight at six months postpartum—this gap could affect BMI calculations.

Since our population was adult women, height was only measured once assuming that majority of participants will have little variation in height in adulthood. We used weight measurements at 6 months rather than 3 months postpartum as 6 months postpartum women’s weight becomes more stable. This is in-line with DHS standards which excludes women less than 3 months postpartum from BMI calculations.

https://dhsprogram.com/data/DHS-Survey-Indicators-Maternal-and-Child-Nutrition.cfm

2. Address Potential Biases in Dietary Intake Assessment
The seven-day food frequency questionnaire (FFQ) is prone to recall bias—consider discussing its limitations.
The FFQ included 50 food items—did it capture seasonal variations in diet?
Explain whether FFQ responses were validated against any other dietary intake methods (e.g., 24-hour recall).

The FFQ did not explicitly capture seasonal variation in the tool (aka ask about certain seasonal foods like some more detailed FFQs would capture), but as described in the methods the FFQ captured food intake at 3 different time periods (3 months, 6 months, and 12 months postpartum), therefore we are able to better capture seasonal variation. In a different analysis we looked at seasonal variation of the food variety score and showed that there was little seasonal variability in this score.

We modified the following sentence on **lines 175-177** to acknowledge seasonal variation.

“Diet in Bangladesh can vary seasonally; therefore, to better capture usual intake, we averaged dietary data of non-starchy staple foods from three, six, and 12 months postpartum.”

Limitations of the FFQ are discussed in the limitations section of the discussion on **lines 385-387**

“Moreover, the dietary tool we used to assess diet quality did not measure quantity and was restricted to a set number of foods. Therefore the diet quality indicators included in the models did not take into account how much of each food item women were eating.”

The FFQ responses were not validated against any other dietary intake methods, but food items listed were selected using formative research from the study population.

Citation:
3. Clarify Food Security Measurement
The five-point Likert scale for food security assessment lacks a clear interpretation—how were responses categorized (e.g., food secure vs. insecure)?
Explain whether the nine-question food security survey aligns with any established food security measurement tools (e.g., Household Food Insecurity Access Scale).

In the statistical analysis section we describe on **lines 194-197** how responses to food security were categorized

“For household food insecurity scores, we summed the scores of the nine questions regarding food access and categorized households into three main categories: food secure (9 points), mild to moderate food insecurity (10-15 points), and severe food insecurity (>15 points) (30). “

This food security tool was developed specifically for this research study site. We have now cited the primary paper for the tool validation.

Na, M., Gross, A.L. & West, K.P. Validation of the food access survey tool to assess household food insecurity in rural Bangladesh. *BMC Public Health* **15**, 863 (2015). https://doi.org/10.1186/s12889-015-2208-1

4. Improve Clarity on Geospatial Data Collection
The study collected household GPS coordinates in 2018–2020 but conducted a geospatial landmark survey in December 2020—why the time gap?
Define how proximity to markets/variety stores was analyzed—was distance used as a continuous variable, or were categorical thresholds applied?
Consider potential errors in GPS data collection, especially in rural areas where accuracy may be lower.

The time gap occurred because of COVID-19 lockdowns. We have added a sentence in the methods on **lines 118-119** to clarify this.

“A gap between geospatial data for households and for landmarks occurred due to restrictions related to COVID-19.”

Proximity to markets and variety stores (now termed grocery stores for consistency with other analyses and discussions with field staff) was defined in the statistical analysis section on **lines 198-205.**

“We used density of markets and grocery shops around a woman’s home as proxy measures for a woman’s external food environment (41). To measure the density of food vendors, we imported geospatial data into ArcPro and projected coordinates to EPSG: 3106 (Gulshan 303 Bangladesh Transverse Mercator) to calculate buffer areas around each household in meters. For each household, we defined the density of markets as the number of markets within 1600m of a household and the density of grocery shops as the number of grocery shops within 400m of a household. We chose these boundaries to reflect density at the village level for markets and immediate proximity for grocery shops (42). For this analysis, we categorized the density of markets and grocery shops using quartiles based on the distribution of data.”

5. Justify Market and Variety Store Definitions
The definition of markets (≥5 permanent shops open daily) and variety stores (standalone vendors selling groceries) seems arbitrary.
Explain how these definitions impact findings—did proximity to these markets correlate with nutritional outcomes?

These definitions are based on a geospatial landmark assessment conducted by the study team. Definitions were defined by the field team with local knowledge and context. In the results section on **lines 244-290** we describe how these findings correlate with nutritional status. For example, on **lines 276 to 279** we state

“Compared to postpartum women living in households with ≤2 markets within 1600m of their household, women who lived in households with >6 markets within 1600m had a 28% (RRR: 0.72; 95% CI: 0.55-0.95) reduction in risk of being classified as underweight (p=0.02) (Figure 2 & Supplemental Table 3).”

Overall Suggestions
Ensure consistency in anthropometric data collection to minimize errors.
Acknowledge recall bias in dietary assessment and justify the FFQ approach.
Provide more detail on geospatial analysis, including how spatial relationships were assessed.
Clarify and justify definitions of food security and market access to ensure meaningful interpretations.

The result tables are not understandable.

Thank you again for your thorough review. We hope that our edits (described above) further clarify our methodology. In addition, I apologize that the tables were not understandable. PLOS Global Public Health asks that tables be embedded in the text. For this reason, tables may split pages in the word/pdf document sent to reviewers. We will ensure that this does not happen when the paper is formatted for publishing.

# Reviewer #2:

Overall, the manuscript provides an interesting exploration of geospatial variation in risk factors that adds to the literature on population nutrition.

Thank you so much for taking the time to review our manuscript. We believe that your review and the changes we made to address your comments have significantly improved the manuscript. We have attempted to address each comment in detail below. Line numbers refer to the line numbers in the clean manuscript.

General comment (abstract conclusions, discussion, etc): The study population is post-partum women. Given that you are unable to adjust for pre-pregnancy weight or weight retention, suggest caution in generalizing conclusions beyond this population. Suggest that sentences summarizing the evidence – including the conclusion statement of the abstract – clearly state that findings are among post-partum women.

Thank you for this comment. We agree it is unfortunate we were unable to adjust for pre-pregnancy weight or weight retention, which is also noted in our limitations section of the discussion. We have reviewed the abstract and discussion to ensure that we clearly state that our findings are among post-partum women.

Abstract: Please add additional details on analytic methods for assessing geospatial variation to the abstract (methods section).

Thank you for this suggestion. We have added this to the text in the abstract on **lines 8-9.**

“We assessed geospatial variation in nutritional status at the individual level using point pattern analysis and at the spatial area level using global Moran’s I.”

Methods: Were all women who were enrolled in the Protein Plus trial eligible for this analysis regardless of birth outcome?

Women would not have been approached for the Protein Plus trial unless they had a live birth and the infant survived until 3 months of age. We have added a citation to the main trial paper on **lines 79-80**.

“Details of the Protein Plus trial are described in detail elsewhere (29).”

Methods: The parent study supported postnatal care (including reminders). Did you explore/account for difference across study arms?

mCARE-II (the parent study) tested whether a digital health intervention package could improve neonatal and perinatal survival. The mHealth package of interventions includes workflow scheduling for guided household registration, census, pregnancy surveillance, and antenatal, postnatal, and essential newborn care visit reminders. We do not believe this would likely have a significant impact on maternal weight 6 months post-partum.

Methods: From the dataset it appears that weight at 3 months post-partum was available. Was weight at enrollment in the mCARE-II trial also available? Is there a reason that these longitudinal weight measurements were not included in the models?

Thank you for this comment. As a research team, we had several discussions over which weight variables to use. Unfortunately, we did not have weight available at enrollment in the mCARE-II (early pregnancy). DHS excludes women less than 3 months postpartum from BMI calculations, therefore we did not calculate BMI at 3 months in our analysis either.

https://dhsprogram.com/data/DHS-Survey-Indicators-Maternal-and-Child-Nutrition.cfm

Methods: Anthropometric data quality can impact evaluation of nutritional status. Often different enumerators are assigned to different geographic areas of the study catchment area in a way that could impact analysis of geospatial clustering. Can you describe what steps you took to explore anthropometric data quality and rule out the possibility that different quality achieved by enumerators confounded the results.

The interviewers doing anthropometry are highly trained and have years of experience. They did a refresher training with the field team research manager on standardized protocols and then went through the standardization process with someone from the quality control team serving as the gold standard. In this training they assessed inter- and intra-observer technical error of measurement. They had to meet 1.0% and 1.5% for inter-observer and intra-observer TEM, respectively. There was regular supervision by the QC team. We have added additional information in the main text on **lines 102-105**.

“Prior to data collection, field workers received a refresher training on standard anthropometry protocols and were required to achieve a target of 1.0% and 1.5% for inter-observer and intra-observer technical errors of measurement, respectively. Additionally, field workers received regular supervision from the quality control team throughout data collection.”

Results: You report that 14.2% of the sample were missing weight which is a reasonably high proportion. Supplemental figures – helpfully - describe demographic characteristics of the sample of individuals included v. excluded from analysis (primarily due to missing weight). However, can you speak to whether there was an geospatial clustering of individuals excluded from analysis?

This is an interesting point and important point. While we did not do a formal test of clustering for the missing data, we did look at a map of the missing data to see if missing data were evenly distributed across the study area. We found an even distribution of missing data across the research site. In addition, due to the large sample size and population density of the area all regions of the study area were well represented in the analysis.

Results: The authors explore the relationship between individual risk factors and community risk factors with nutritional status. However, it seems that there is a unique opportunity to explore the extent individual covariates of interest geographically cluster in a way that impacts nutritional status. For example, you find that LSI is associated with nutritional status but is living in a poorer neighborhood (cluster median LSI) independently associated with nutritional status? What about neighborhoods that are on average younger and/or less educated?

While conducting our analysis, we also thought this might something interesting to explore. We ran bivariate choropleth maps to see if community averages of covariates clustered in a way that impacted nutritional status. I have included the bivariate map for LSI. We ended up not including these in this paper because the only clear pattern we saw was with LSI. Since LSI was such a strong indicator in the multinomial analysis we were not entirely sure this bivariate analysis added anything new to the paper and therefore decided to exclude (particularly because this bivariate analysis cannot control for other risk factors).

Results: Paragraph beginning on line 282 includes a lot of double negative phrasing. Consider revising.

Thank you for this suggestion. We have revised a few sentences to avoid double negative phrasing.

Results: Supplementary Figures 2 and 10 - Semivariogram for Maternal BMI and associated residuals appear identical (except for the title). Can you confirm that the correct figures are included.

I have double checked the code for these figures and while they do look very similar these are indeed plots generated from different data. Data from Supplemental Figure 2 is from BMI, while Supplemental Figure 10 is model residuals. After re-running the analysis after excluding women with greater than 10kg weight change, these figures look slightly less identical than previously.

Results/Dataset. Assuming that m3weight and m6 weight refer to weight at 3 and 6 months post partum respectively, there are many women with implausible changes in weight (and therefore BMI). To use an extreme example, there are women with 32kg losses and 38kg gains over a 3-month time period. Over 1% of the sample having +/-6kg weight change over 3 months. Please discuss what data quality reviews (particularly for the anthropometric data) were performed. How would exclusion of implausible values impacts findings?

Thank you for raising this issue. In our original analysis, we had flagged that there were some women (n=18) with greater than 10+kg weight change. Due to the small sample size we opted to included these women in the original analysis. The extreme examples noted in the comment were excluded in the original analysis based on the inclusion criteria. Given your comment, we discussed this among co-authors and have decided to exclude women with greater than 10kg weight change in the analysis. This had a minimal impact on our findings. Results changed slightly (typically either by 1 or 2 decimal points) and the overall results stayed the same.

Discussion: The lack of spatial variation at the household level given clear variability in community-prevalence is the key and somewhat surprising finding. To what extent does this have to do with parameterization (e.g., the use of the continuous v. categorial variable). Do you see the same effect if you look at mean BMI for an area rather than prevalence? Do we see the same phenomenon in previous literature? To give confidence that these findings are not spurious consider: (1) adding figures similar to supplemental Figures 6 and 7 but with mean BMI, (2) including additional documentation in the results on model fit / validation.

Yes, this is a great point. We have reorganized the text in the discussion section to better highlight this finding and present our hypothesis on why we found spatial variation at the area level but not the household level. We do not believe this is parameterization because we conducted a point pattern analysis to looked at spatial clustering using categories of BMI (Supplemental Figures 3 and 4) and found no spatial clustering at the household level.

On **lines 316-322**, we state:

“Within the JiVitA study site, we found geospatial variation in nutritional status at the area level, although at the household level, we found no evidence of geospatial variation. The lack of statistically significant spatial dependence at the household level is most likely due to high population density and variability in nutritional status among neighboring women within the same area unit.”

In addition we added text to the discussion about two studies who found geospatial variation in maternal health indicators at the area level in Bihar, India and Afghanistan. To our knowledge, we have not found a study that has reported spatial variation at the household level for maternal health indicators. This text has been added on **lines 326-330**,

“For example, in Bihar, India, a study found that trends in several reproductive, maternal and child health indicators varied spatially at the block-level during a state-wide scale-up of a maternal and child health intervention (27). For example, in Bihar, India, a study found that trends in several reproductive, maternal and child health indicators varied spatially at the block-level during a state-wide scale-up of a maternal and child health intervention (27). Moreover, a nationally representative analysis in Afghanistan found geospatial variation at the province level with higher prevalences of underweight for women of reproductive age in the north and northeast provinces (48).”
