# Supplementary material for: Geospatial variation and risk factors for malnutrition among postpartum women in rural Bangladesh
Source: PLOS Glob Public Health. 2026 Feb 6;6(2):e0005726. doi: 10.1371/journal.pgph.0005726 (PMC12880669; doi:10.1371/journal.pgph.0005726)
Supplement: Attachment — Submitted filename: Comments-PLOS.docx [file pgph.0005726.s004.docx]

**Comments**

This is generally are good paper that tries to establish proximate factors affecting spatial variations in nutritional status among postpartum women in rural Bangladesh. I just have a few comments for the authors to improve the paper:

1. I think the literature for such an important paper could be improved much further. The authors have something on dietary intake as a proximate factor. But they are quite silent on another major proximate determinant in the area of diseases and infections, such as Diarrheal diseases, Respiratory infections (e.g., pneumonia), Malaria, Intestinal parasites (worms) and HIV/AIDS. In fact, Pubmed, Biomed, PLOS etc have lots of publications even in Bangladesh on this area that the authors could use to enrich the literature.
2. *Lines 159-161*: What is the justification for the use of K-functions instead of other functional methods? It will also be helpful to show the K(d) curve output of the estimation.
3. *Lines 185*-*187*: The use of market density and grocery shop density as proxies for food environment needs some further explanation to understand the rationale behind it.
4. *Lines 211-213*: Are the items used for estimating the food insecurity score coming from a validated tool? Give a reference, please. Also, what is the coefficient of reliability?
5. *Lines 218*-*219*: What informed these cut inputs 1600m for markets and 400m for grocery shops?

*Samuel Kojo Antobam*
