# Supplementary material for: Geospatial variation and risk factors for malnutrition among postpartum women in rural Bangladesh
Source: PLOS Glob Public Health. 2026 Feb 6;6(2):e0005726. doi: 10.1371/journal.pgph.0005726 (PMC12880669; doi:10.1371/journal.pgph.0005726)
Supplement: Attachment — Submitted filename: Response to Reviewer Round 2.docx [file pgph.0005726.s005.docx]

**Comments**

This is generally are good paper that tries to establish proximate factors affecting spatial variations in nutritional status among postpartum women in rural Bangladesh. I just have a few comments for the authors to improve the paper:

Thank you for taking the time to review this paper. We really appreciate your feedback and have made efforts to address each of your comments. Our responses are listed below in green.

1. I think the literature for such an important paper could be improved much further. The authors have something on dietary intake as a proximate factor. But they are quite silent on another major proximate determinant in the area of diseases and infections, such as Diarrheal diseases, Respiratory infections (e.g., pneumonia), Malaria, Intestinal parasites (worms) and HIV/AIDS. In fact, Pubmed, Biomed, PLOS etc have lots of publications even in Bangladesh on this area that the authors could use to enrich the literature.

Thank you for this comment, but in this paper we most interested in determinants associated with the double burden of malnutrition which is why our risk factors and literature focused on dietary intake, food security, food environment, wealth, and educational level. We have noted in the introduction and discussion that this focus is also supported by other relevant studies.

2. *Lines 159-161*: What is the justification for the use of K-functions instead of other functional methods? It will also be helpful to show the K(d) curve output of the estimation.

Thank you for this question. In this analysis, we are trying to assess spatial clustering of both underweight and overweight/obesity with women who are classified as normal weight acting as controls. In supplementary tables figures 3 and 4, we show the difference in k functions between cases (underweight or overweight) and controls (normal weight). This is more informative than comparing the k(d) curve to the line of complete spatial randomness, as human settlements are often clustered. We have also included references to justify the use of the difference in K-functions (reference 36 & 37).

To make this more clear in the text we have revised the text the as follows on lines 147-149:

“In addition, we created BMI categories and we conducted a point pattern analysis. We estimated spatial intensity of underweight and overweight (expected number of events per unit area) to map out the concentration of cases (underweight/overweight). To characterize smaller-scale spatial variation in the BMI categories, we calculated the difference in K functions to assess whether women who are categorized as underweight based on BMI cluster more (are more spatially compact) compared to women categorized as normal weight and repeated for the overweight to normal weight comparison (36,37).”

And on Line 241-245.

“When assessing geospatial variation at the household level, we found no evidence of spatial dependence for BMI either as continuous measure (BMI semivariogram was relatively flat) or categorical (difference in k functions were within confidence bands) **(Supplemental Figures 2-4).** Although the spatial concentration of women classified as underweight revealed an east west gradient, this was not consistent with spatial concentration in overweight cases **(Supplemental Figures 5 & 6)**.”

3. *Lines 185*-*187*: The use of market density and grocery shop density as proxies for food environment needs some further explanation to understand the rationale behind it.

Thank you for this feedback. We selected market density and grocery shop density as proxies for the food environment after conducting an analysis look at relationship between food environment and dietary intake in the same study area. In addition, density is a proxy for availability of vendors which has found to be more consistently associated with nutritional status. We have added the following text on lines 207-209 based on your feedback.

“We selected density measures as proxy of food environment because of previous analysis found a relationship between density of markets and dietary intake (38). In addition, the density of food vendors rather than the distance to food vendors has been found to be associated with nutritional status (42,43).”

4. *Lines 211-213*: Are the items used for estimating the food insecurity score coming from a validated tool? Give a reference, please. Also, what is the coefficient of reliability?

We have included the reference “Na M, Gross AL, West KP. Validation of the food access survey tool to assess household food insecurity in rural Bangladesh. BMC Public Health. 2015 Dec;15(1):863.” This tool was developed and validated in the same study region. For clarity, we have added the sentence on line 114.

“To measure food security, field staff asked participants a series of nine questions about how often they experienced challenges with food access in the prior six months using a five-point Likert scale (1=Never, 5=Most days of the week). Questions on food security were from a validated survey tool developed in the same study area (30).”

5. *Lines 218*-*219*: What informed these cut inputs 1600m for markets and 400m for grocery shops?

These boundaries were selected based on previous literature that had used similar cutoffs to reflect village level availability (1600m) and immediate availability (400m). We have also added an additional citation that used similar boundaries as well for a study in India.
